# Safety Evaluation of EXPAREL (DepoFoam Bupivacaine) Administered by Repeated Subcutaneous Injection in Rabbits and Dogs: Species Comparison

**DOI:** 10.1155/2011/467429

**Published:** 2011-10-05

**Authors:** Brigitte M. Richard, Douglas E. Rickert, Paul E. Newton, Laura R. Ott, Dean Haan, Abram N. Brubaker, Phaedra I. Cole, Paul E. Ross, Marlon C. Rebelatto, Keith G. Nelson

**Affiliations:** ^1^Department of Toxicology, Clinical Research & Drug Safety Assessment, Pacira Pharmaceuticals Inc., San Diego, CA 92121, USA; ^2^Pharmacokinetics, Mill Ridge Road, Raleigh, NC 27613, USA; ^3^Toxicology Division, MPI Research Laboratories, North Main Street, Mattawan, MI 49071, USA

## Abstract

EXPAREL (bupivacaine extended-release liposome injection), DepoFoam bupivacaine, is in development for prolonged postsurgical analgesia. Repeat-dose toxicity studies were conducted in rabbits and dogs to compare the potential local and systemic toxicities of EXPAREL and bupivacaine HCl (Bsol), and the reversibility of any effects. Dogs tolerated much larger doses than rabbits. EXPAREL-related minimal-to-moderate granulomatous inflammation was noted at the injection sites. In recovery animals, the granulomatous inflammation was observed less frequently and was characterized by an increased number of multinucleated giant cells. These effects were considered a normal response to liposomes and nonadverse. Rabbits are more sensitive than dogs. In rabbits, convulsions were noted with EXPAREL and more frequently with Bsol; a NOAEL was not identified. In dogs, EXPAREL was well tolerated (NOAEL > 30 mg/kg/dose). The cumulative exposure of EXPAREL in these studies is well in excess of the proposed maximum single-dose exposure that is intended in humans.

## 1. Introduction

The investigational drug EXPAREL (DB, DepoFoam bupivacaine; bupivacaine extended-release liposome injection) is a multivesicular liposomal formulation of bupivacaine being developed for postsurgical analgesia ([1], Angst 2006). Dierucoylphosphatidylcholine (DEPC), a phospholipid excipient, is unique to the delivery system present in EXPAREL and has not been previously included in other DepoFoam-based approved products, that is, DepoDur (morphine sulfate) and DepoCyt (cytarabine). 

Since the association of bupivacaine to multivesicular liposomes delays the vascular absorption of bupivacaine released from the lipid vesicles, DepoFoam bupivacaine may prevent accumulation of unexpectedly high (possibly toxic) blood and/or tissue concentrations of bupivacaine compared to bupivacaine HCl (Bsol) and therefore may provide a safer alternative to current therapies. 

Systemic reactions to bupivacaine mainly involve the central nervous (CNS) and/or cardiovascular (CV) systems [2]. These effects have been described in the literature in several species including humans [3–8]. (Since these effects have been extensively summarized in the literature, only a selection of relevant references is given here (please refer to the Discussion section for further detail).) 

When given in sufficiently large doses, liposomal formulations may be irritating to subcutaneous (sc) tissues, causing nonspecific local reactions. Particularly during repeated exposure, the presence of exogenous lipid materials in the sc space may serve as a nidus for the development of a foreign body type reaction in surrounding tissues. Therefore, it seems possible that prolonged, repeated exposure to EXPAREL could intensify the degree of sensitivity to bupivacaine and/or DepoFoam particles particularly in rabbits, because of the thinness of the skin layer and relative absence of sc fat.

As part of the nonclinical development program, the safety of repeat-dose administration of EXPAREL compared to Bsol was evaluated in two species in accordance with International Conference on Harmonization (ICH) guidelines. 

These multiple-dose studies in rabbits and dogs (nonsurgical model) were designed to complement single-dose toxicology testing (surgical hernia repair model) in the same species, in which animals were exposed to the same amount of drug. 

Groups of animals were given EXPAREL at a dose level of 9 mg/kg, 18 mg/kg, or 30 mg/kg in comparison with Bsol 9 mg/kg (7.5 mg/mL), or saline via sc twice weekly injection. These studies included evaluation of both local effects as well as the usual broad range of systemic effects. 

It was possible to meaningfully compare the toxicology findings and concurrent systemic exposure in rabbits and dogs since the same protocol in a whole-body system, assay methodology, and data acquisition systems were used. The clinical relevance of the toxicology results was evaluated in relation to the intended clinical use of EXPAREL (single dose) in patients.

## 2. Materials and Methods

### 2.1. Materials

#### 2.1.1. Description of DepoFoam^TM^ Technology

The DepoFoam drug delivery system is a proprietary, injectable technology that provides a sustained release of therapeutic compounds. The DepoFoam system consists of microscopic, polyhedral, lipid-based particles composed of numerous nonconcentric, aqueous chambers containing the drug in solution. Each chamber in this multivesicular liposome is separated from adjacent chambers by lipid membranes [9, 10].

The DepoFoam particle components are naturally occurring or synthetic analogues of common lipids, including phospholipids, cholesterol, and triglycerides. 

#### 2.1.2. Test Article

EXPAREL (DepoBupivacaine, DB; bupivacaine extended-release liposome injection using multiv-esicular DepoFoam technology), 15 mg/mL and 25 mg/mL (expressed as anhydrous bupivacaine base), was provided by Pacira Pharmaceuticals, Inc., San Diego, Calif. In the higher-concentration EXPAREL system (25 mg/mL), the multivesicular liposome particle concentrations were increased from approximately 35–40% of the total suspension volume, to approximately 60–70% total suspension volume. (This provided maximum local concentrations at the injection site.) 

#### 2.1.3. Reference Product

Sensorcaine-MPF (methyl paraben free; bupivacaine HCl injection, USP, Bsol 0.75%) was supplied by AstraZeneca, Wilmington, Del. 

#### 2.1.4. Control Article

Saline (0.9% sodium chloride injection USP) was supplied by Abbott Laboratories, North Chicago, Ill. 

#### 2.1.5. Animals

New Zealand White rabbits and Beagle dogs were supplied by Covance Research Products, Kalamazoo, Michigan, and Marshall BioResources, North Rose, NY, respectively. The animals were 5–8 months (rabbit) and 4 months (dog) of age on arrival. The animals were acclimated for a period of at least one week. The animals received LabDiet (Certified Rabbit Diet no. 05007 and Certified Dog Diet no. 05322; PMI Nutrition international, Inc., Richmond, Ind).

### 2.2. Methods

#### 2.2.1. Study Protocol

All protocols were reviewed and approved by the Institutional Animal Care and Use Committee (IACUC) of MPI Research, Inc., Auxvasse, Mo, for compliance with regulations prior to study initiation. These studies were conducted according to ICH guidelines and in accordance with Good Laboratory Practices principles as set forth by the United States Food and Drug Administration (FDA), 21 CFR Part 58.

The repeat-dose studies were designed to use the fewest number of animals possible, consistent with the objective of the studies, with particular consideration to eliminating the impact of surgical intervention on the normal behavior or pattern of study animals.

#### 2.2.2. Rationale for Dose Regimen

Groups of animals (*N* = 3/sex/group) were given EXPAREL at 9, 18, or 30 mg/kg/dose of a more highly concentrated formulation (bupivacaine 25 mg/mL, with the proportional increase in lipid concentrations), Bsol 9 mg/kg/dose (7.5 mg/mL), or saline via sc twice weekly injection. Each dose was administered by bolus injection.

The protocols were designed to assess any exaggerated pharmacological response and potential local and systemic toxicities by selecting dose levels and concentrations at multiples higher than the intended therapeutic dose. The sc injection route administration was considered appropriate as an alternate route of delivery to simulate the wound infiltration route in the clinic. 

The dose levels and volumes were selected on the basis of available data from proprietary single-dose studies in rabbits and dogs (hernia repair model), maximum projected clinical dose, and published literature discussed here.

The selection of the highest dose is based on an EXPAREL dose of 30 mg/kg given up to the limits of solubility (25 mg/mL). For the 30 mg/kg dose, the injection volume of 1.2 mL/kg was calculated based on the supplied concentration of 25 mg/mL. It should be noted that the studies were not designed to study specifically volume effects. Greater volume of more concentrated drug was necessary to achieve the highest dose level of EXPAREL 30 mg/kg. Smaller volumes or more dilute suspension of bupivacaine were used at lower EXPAREL dose levels. 

Both the highest volume delivered (1.2 mL/kg) and the highest dose administered (9 mg/kg) of bupivacaine HCl were evaluated. 

The 9 mg/kg dose was based upon the published intr-avenous (iv) lethality of 5–11 mg/kg for bupivacaine [11], and the lethality seen in a previous study in rabbits conducted in the same laboratory (data not shown). Based on these results, the maximum total nonlethal bupivacaine dose was ~9 mg/kg or 1.2 mL/kg of bupivacaine HCl (7.5 mg/mL). 

#### 2.2.3. Rationale for Dose Frequency

The dose sites were alternated between two scapular regions so that the studies could be performed without the potential concern of injection site irritation obscuring or otherwise compromising the ability to discern systemic effects resulting from treatment-related observations. Specifically, the twice weekly doses were rotated at two different sites to the right of the dorsal midline (site 1) and to the left of the dorsal midline (site 2). The borders of the sites on opposite sides of the midline had at least two inches to ensure that there was no cross contamination between the sites. The dose was administered on Days 1, 8, 15, and 22 (site 1) and on Days 4, 11, 18, and 25 (site 2). An individual site was dosed once every 3 days. 

The study design takes into consideration the slow egress of the lipid components from the injection sites as previously shown in research studies (data not shown). The repeat dose administration studies with EXPAREL were designed with an intermittent dosing schedule to allow enough time for dose egress from the injection sites between each dose administration and minimize the risk of plasma drug accumulation while providing sufficient exposure. The twice weekly dosing schedule (Days 1, 4, 8, 11, 15, 18, 22, and 25) was selected based on computer-based simulations using WinNonlin (assuming linear kinetics) which suggested continuous exposure of bupivacaine with no or minimal accumulation over the course of the study. The simulated profile was derived from actual single dose data (high dose only) and extrapolated to twice weekly administration (data not shown).

Following 25 days of administration, three anima-ls/sex/group were maintained for a 4-week treatment-free recovery period. At the end of the dosing (Day 25) and recovery period (Day 50), animals were sacrificed (*N* = 3/sex/group/period).

#### 2.2.4. Endpoints

Endpoints included clinical signs, clinical pathology, electrocardiographic recording (EKG, dog only), organ weight, full histopathologic tissue evaluation, and toxicokinetics on Day 1 (first dose) and Day 25 (last day of dosing) (through 72 hours postdose). EKG was performed prior to dosing and during the last week of dosing (Day 22) and the last week of the recovery period (Day 50). 

 Any observable involuntary movements were noted. Special attention was paid to signs of CNS disturbances and seizures (i.e., tonic/clinic convulsions, tremors) 

Microscopic examination of fixed hematoxylin and eosin-stained paraffin sections was performed on the sites of injection and other protocol-specified tissues. A four-step grading system was used to define gradable lesions for comparison between dose groups (i.e., minimal, mild, moderate, and severe). 

#### 2.2.5. Pharmacokinetic Assessment

Toxicokinetic samples were collected from the 3 animals/sex/group designated for a 4-week recovery period. Blood samples were taken from the jugular veins in these groups on Day 1 and on Day 25, at 0 (predose), and at 0.5, 1, 2, 4, 8, 12, 24, 48, and 72 hours after dosing. Samples were placed in tubes containing K_3_ EDTA and stored on an ice block until centrifuged. Each sample was 0.5–1 mL. The test animals were not fasted before blood collection unless collection times coincide with clinical pathology collections. The plasma samples were stored frozen at approximately −70°C until analyzed.

Plasma concentrations of bupivacaine were measured by MPI Analytical, Mattawan, Mich, using a validated LC-MS/MS method. The assay is selective for the quantification of bupivacaine in rabbit and dog K_3_EDTA plasma in the concentrations ranging from 10.0 to 10,000 ng/mL. The PK parameters were evaluated by a noncompartmental model using WinNonlin, version 5.0 (Pharsight Corp., Mountain View, Calif). The PK parameters were maximum plasma concentration (*C *_max_), time at which the *C *_max_ occurred (*t *_max_), and area under the plasma concentration, time data (*AUC *_0-t_). The half-life (*t *_1/2_) was calculated in the late phase of plasma concentration versus time curve. 

## 3. Results and Discussion

### 3.1. Toxicology Results in Rabbits

There were no test article-related effects on body weight, food consumption, hematology, coagulation, clinical chemistries, urinalysis, or organ weight endpoints.

One female died on Day 19 one day after receiving the sixth dose of EXPAREL (30 mg/kg). In the last scheduled observations, the animal was normal. Microscopically, no cause of death was determined. In addition to the changes seen at the injection sites in all the EXPAREL groups (i.e., moderate swelling/thickening of the injection site), this animal presented with microscopic findings consisting of splenic, lymph node, and thymic lymphoid depletion. This stress-associated lymphoid depletion is a common finding in animals that die on study and is associated with physiological stress. Additionally, a small amount of material consistent with food matter was seen in the lungs, due most likely to perimortem aspiration as there was no associated inflammation. It should be noted that as a result, since this rabbit was normally part of the recovery group, there were only two of the three females surviving though the recovery period.

When comparing with the same dose of EXPAREL, Bsol (9 mg/kg) was associated with a more frequent incidence of tremors/convulsions (3/3 males and 1/3 female after the third dose and 1/3 male after the fifth dose) (Table 1). While convulsions were not seen at the 30 mg/kg dose of EXPAREL, the convulsions seen with EXPAREL at the 9 mg/kg (1/3 male after the first dose) and 18 mg/kg (1/3 female and 1/3 male after the fourth and fifth dose) were considered to be bupivacaine-related because of the convulsions seen in the Bsol dose group.

Macroscopically (Day 26), increased incidence of mild-to-moderate red discoloration and swelling/thickening of both of the injection sites was noted in animals of both sexes at the 30 mg/kg dose of EXPAREL. Red discoloration and swelling/thickening were also noted at a low incidence in the 9 and 18 mg/kg dose groups, but these local reactions were comparable to Bsol or control saline group. 

In recovery animals, mild red discoloration was seen at a low incidence in all three EXPAREL-dosed males at all the dose levels and in 1/3 female receiving EXPAREL 30 mg/kg. These changes were, for the most part, correlated with the microscopic findings of hemorrhage (HEM) and neovascularization (NV) outlined below. 

**Figure 1:**
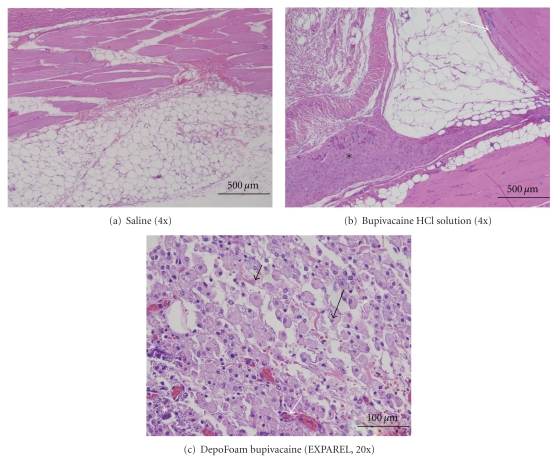
Injection Site Findings in Rabbits on Day 26. (a): Saline Control. H&E 4x, (b): Bupivacaine HCl Solution, 9 mg/kg. H&E 4x, (c): DepoFoam Bupivacaine 30 mg/kg H&E 20x. Annotations are as follows: Black arrows: vacuolated macrophages, White arrows: neovascularization, black asterisks: inflammation (chronic-active to chronic), white asterisks: mineralization.

Test article-related microscopic findings (H&E staining) were seen in both the sc injection sites, at all dose levels of EXPAREL in the majority of animals after repeated dosing. Microscopic findings consisted primarily of HEM, NV, vacuolated (foamy) macrophages (VMs), and inflammation (chronic-active or subacute). There was no consistent dose-dependent response seen, except in increased numbers of VMs at higher dose levels. This finding consisted of individual VMs with finely vacuolated, foamy cytoplasm forming aggregates, and/or extending along fascial planes, with rare small numbers of lymphocytes and/or plasma cells and no significant numbers of multinucleated giant cells (GCs). All findings were minimal-to-mild/moderate. None of the changes above were seen in either saline or Bsol group (Figure 1).

Injection site lesions resolved to some degree at recovery, although minimal to mild HEM, VMs, NV, and inflammation were all present in low numbers in recovery animal. The presence of VMs appeared to resolve in a dose-dependent manner with none seen in males at the 9 mg/kg dose or in females at the 9 or 18 mg/kg dose of EXPAREL. 

### 3.2. Toxicology Results in Dogs

There were no test article-related effects on clinical observations, body weight, food consumption, hematology, coagulation, clinical chemistries, urinalysis, or organ weight endpoints. There were no EKG abnormalities caused by EXPAREL, Bsol, or saline.

When compared to Bsol or saline control group, the only notable findings were EXPAREL-related macroscopic and microscopic observations in the injection sites of both male and females (Days 26 and 54).

Swollen or thickened injection sites were noted in a low number of terminal and recovery animals. Often, there was no microscopic correlate to the red discoloration at the injection sites. In rare occasions, the red discoloration corresponded with HEM, edema, and/or sc subacute inflammation. This macroscopic finding was considered to be a result of physical trauma from the injection procedure and not associated with treatment.

**Figure 2:**
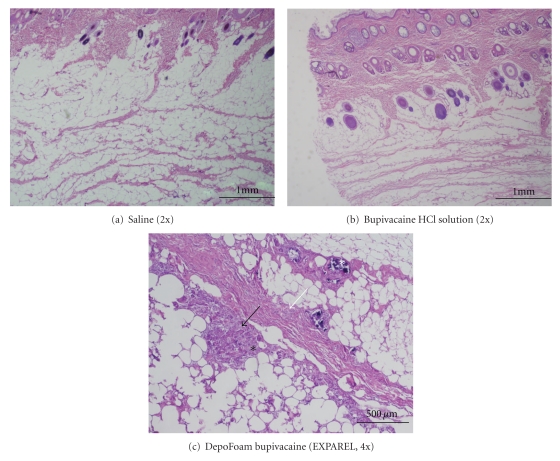
Injection site findings in dogs on day 54. (a): Saline control. H&E 2x, (b): bupivacaine HCl solution, 9 mg/kg. H&E 2x, (c): DepoFoam Bupivacaine 30 mg/kg H&E 4x. Annotations are as follows: black arrows: vacuolated macrophages, white arrows: neovascularization, black asterisks: inflammation (chronic-active to chronic), white asterisks: mineralization

On both Day 26 and Day 54, minimal-to-moderate Gi was observed in the sc tissue of male and female dogs. Similar microscopic findings were not observed in Bsol or saline control group (Figure 2).

On Day 26, Gi was characterized by numerous VMs and fewer lymphocytes, plasma cells, and/or GCs formed by fused Macs with abundant cytoplasm and nuclei scattered irregularly throughout the cytoplasm. The Gi was commonly associated with edema and/or mineralization. The mineral deposits were commonly surrounded by GCs. On Day 54, Gi was observed less frequently and was characterized by an increased number of GCs sometimes associated with mineralization, but not edema. In one male receiving EXPAREL 9 mg/kg, minimal edema not associated with inflammation was noted. 

In the EXPAREL groups, minimal-to-mild signs of hemorrhage, acute inflammation, erosion, epidermal exudates, and/or subacute inflammation were observed sporadically at the injection site of some terminal and recovery animals. The subacute inflammation was primarily associated with hair follicles and rarely surrounded intralesional mites consistent with Demodex canis. These findings were considered procedural. 

### 3.3. Pharmacokinetic Results

The pharmacokinetic results are shown in Tables 2–4. Species difference was observed with lower *C *_max_ (↓ 4 fold) and AUC (↓ 5 fold) for all dose levels for EXPAREL (rabbit versus dog). The same observation was made for Bsol with lower *C *_max_ (↓ 4-9 fold) and AUC (↓ 4 fold).

Systemic exposure in female rabbits on Day 25 tended to be larger than that in males (data not shown). In dogs, there was not marked or consistent gender difference with regard to the PK parameters for bupivacaine. 

The PK results indicate that rabbits and dogs were exposed to bupivacaine in a dose-related (although not strictly dose-proportional) manner after twice weekly repeated dosing of EXPAREL, at doses ranging from 9 to 30 mg/kg. The degree of accumulation for EXPAREL upon repeat dosing was no greater than two- to threefold which indicate minimal-to-moderate carryover from the previous dose (Tables 2 and 3). 

**Figure 3:**
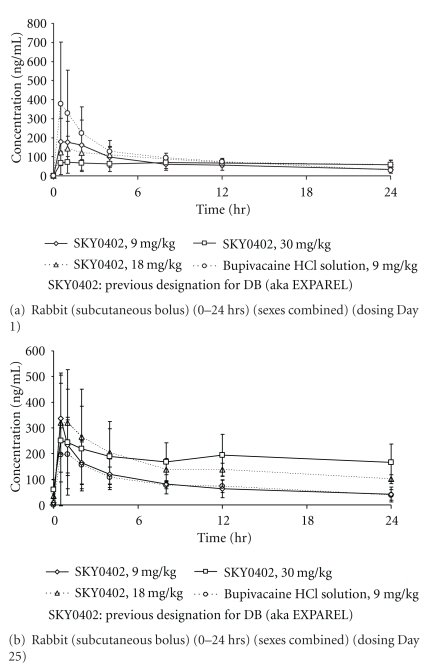
Mean plasma concentrations of bupivacaine following subcutaneous injection of DepoFoam bupivacaine (SKY0402, aka EXPAREL) and bupivacaine HCl solution in rabbits (values represent mean ± SD, *N* = 3/sex/group). SKY0402: previous designation for DB (aka EXPAREL).

**Figure 4:**
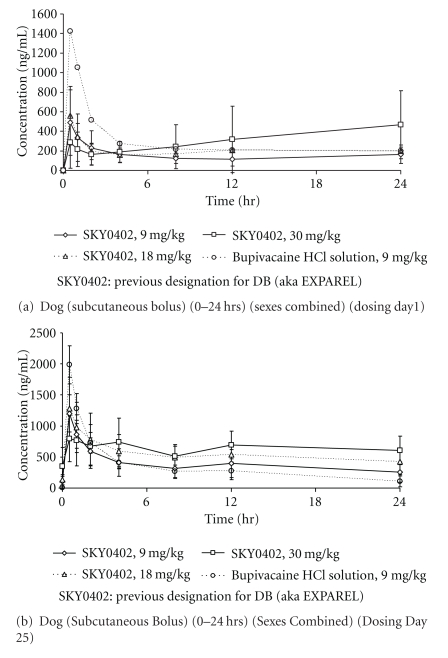
Mean plasma concentrations of bupivacaine following subcutaneous administration of DepoFoam bupivacaine (SKY0402, aka EXPAREL) and bupivacaine HCl solution in dogs (values represent mean ± SD, *N* = 3/sex/group). SKY0402: previous designation for DB (aka EXPAREL).

In both species, the kinetic release profile was consistent with sustained release of the drug from the delivery system at the site of administration (Figures 3, and 4). The attenuation of *C *_max_ was on the order of two- to threefold compared to Bsol after the first dose. The accumulation was more evident at the 30 mg/kg dose in rabbits compared to dogs. 

## 4. Discussion

When interpreting toxicology results, consideration has to be given to the particular sensitivity of the species to local reactions. The rabbit is, as a general rule, more sensitive than other species to the action of most substances [12]. The sensitivity of the rabbit is due to the thinness of the skin layer and the relative absence of sc fat [13]. It is not surprising therefore that a series of twice weekly injections of EXPAREL in which each exposure would progressively intensify the degree of sensitivity may cause local irritation from prolonged tissue exposure. After several repeat injections, the compartments are nearly saturated and therefore may no longer protect against potentially toxic concentrations. 

Also, when assessing the potential existence of cumulative systemic effects of bupivacaine, the resulting plasma kinetics is an important safety consideration because systemic absorption of bupivacaine causing rapid high peaks are associated with a more pronounced risk of CNS and CV effects. A brief review of the literature is provided below.

The toxic response of bupivacaine is characterized by a complex interaction between the CNS and CV systems. 

The response, at least in part, depends on how fast the drug is administered, and the resultant blood/tissue concentrations in target tissues are affected. Particularly, injection of repeated doses of local anesthetics may cause significant increases in plasma levels with each repeated dose due to slow accumulation of the drug or its metabolites or to slow metabolic degradation. It is difficult to determine the relationship between plasma levels and toxicity when the summated response originates from tissues with different sensitivities, for example, cardiac muscles, neural tissues. 

In animals (and humans), adverse effects associated with bupivacaine are generally dose-related and most often are due to acutely high plasma levels resulting from rapid absorption of bupivacaine at the intended site of action, overdosage (i.e., enhanced absorption), diminished tolerance, or unintentional intravascular injection [12, 14, 15].

In humans, dose-limiting effects generally occur more frequently with bupivacaine doses in the higher ranges. Plasma concentrations of bupivacaine ranging from 3 to 5 *μ*g/mL produce a progression of CNS symptoms, including headache and numbness; with increased plasma concentrations, convulsions may occur [7, 16]. In most cases, life-threatening acute toxicity affecting the CNS and/or CV system is not seen until there are sufficiently elevated blood levels. Bupivacaine can cause severe hypotension, respiratory distress, CV collapse, and cardiac arrythmias including ventricular fibrillation which have been responsible for fatalities [17, 18]. Large doses reaching the CNS system can cause brain-stem depression resulting in severe respiratory depression of apnea. In severe cases, cardiac arrest may occur. 

Cardiotoxicity is less easy to study in man, as the clinical signs are not usually seen until the CNS toxicity is marked. However, CV collapse and even death can occur from low dose of bupivacaine without significant CNS toxicity, possibly as a result of the sudden onset of ventricular fibrillation [19, 20]. During ventricular fibrillation and/or hemodynamic instability, bupivacaine may produce severe myocardial tissue hypoxia and acidosis contributing to the overt toxic reactions [21–23]. Bupivacaine causes differential effects on the peripheral vascular resistance, with both vasodilation and vasoconstriction having been reported [24–28]. In addition, factors influencing plasma protein binding (e.g., surgical stress, acid-base status of the patient, systemic diseases which alter protein production, or competition with other drugs for protein binding sites, as well as flow dynamics) may diminish individual tolerance). 

Acute toxicity of bupivacaine has been reported in mice, rats, rabbits, dogs, pigs, sheep, and monkeys. Endpoints studied includes CNS (convulsions) and CVS toxicity (most commonly, ventricular arrhythmias and circulatory collapse), muscle degeneration and regeneration (particularly in rats), and maternal and fetal toxicity during delivery (mostly in sheep) [29–38]. 

Neurotoxicity manifesting as convulsions is a well-recognized complication of the administration of bupivacaine (and structural analogs) in both animals and humans. CNS toxicity is characterized by a two-stage pathophysiologic process. Shivering, muscle twitching, and tremors precede tonic-clonic seizure activity as increased plasma levels of bupivacaine preferentially block inhibitory central pathways, leaving excitatory neurons unopposed. 

Convulsions may occur due to absolute overdose, inadvertent iv injection or because of accidental early tourniquet release in iv regional anesthesia [39]. The seizure is typically of short duration and self-limiting. Respiratory arrest is common because of the lack of muscle control associated with the seizure. Progression to hypoxia, cyanosis, and cardiac arrest may be rapid because of the consequences of increased oxygen consumption of the tonic muscles and respiratory arrest [40]. Physiological changes such as acidosis and decrease of carbon dioxide tension may affect the CNS toxicity of local anesthetics [41]. 

There are a number of limitations to some published in vivo studies since they were performed in anesthetized animals with the complicating effects of anesthesia and surgery, and bupivacaine was administered at toxic doses which did not allow to evaluate the absolute CNS and/or CV effects. Although there is no generally agreed standard model of toxicity, whole-system models are generally considered more clinically relevant than others. However, the data acquired are complicated by PK/PD interactions at different organ system, progressive (gradual) response, and intrinsic control mechanisms. As a result, the dose response may be discontinued and nonlinear [38]. 

CNS effects are generally assumed to precede CV toxicity; this notion was primarily derived from studies over the past several decades comparing doses causing disappearance of pulsative blood pressure and onset of convulsions effects in sheep [38]. This ratio was proposed as comparative measure of CV toxicity. It was suggested that the higher the ratio, the better the safety margin for a given compound. That is, the wider the safety margin between convulsions and CV collapse, the more time there may be for treatment intervention when early signs of toxicity arise. 

In a recent published report, the utility of site-directed delivery systems to differentiate between CNS and CV system effects has been emphasized [38]. The author questioned the “CNS hypothesis” of cardiotoxicity and commented that it may not be correct or, if it is, it may apply only to massive iv overdose and not be sensitive towards the CNS site-selective doses used in close arterial models. In a CNS site-directed carotid arterial infusion studies, bupivacaine was found to be more potent toward direct CNS toxicity and indirect cardiac toxicity than levobupivacaine and ropivacaine; however, there was no remarkable difference between the agents in nonfatal arrhythmogenicity nor did it find fatal arrhythmias [35]. In site-directed coronary arterial infusion studies, direct cardiac effects of bupivacaine, levobupivacaine, and ropivacaine were reported in the sheep [33]. In such model, the time-course of myocardial depression was similar for bupivacaine, levobupivacaine, and ropivacaine in doses that cause no CVS effects in conscious sheep. All these drugs caused abrupt onset fatal dysrhythmias.

In rabbits, the mean convulsive doses (3.6 mg/kg, 0.18 mg/kg/min) and mean lethal doses of bupivacaine (7.6 mg/kg, ~0.38 mg/kg/min) were determined after slow iv infusion of 0.32 mL/min for 20 minutes, or until the animals died. The clinical signs were salivation, tonic and clonic convulsions, and respiratory arrest. After eight injections of bupivacaine given sc at 30-minute intervals, a dose of 6 mg/kg produced convulsions in 2/5 rabbits while no effects were seen at 5 mg/kg [14]. Metabolic consequences of seizures include acidosis, hypoxia, and hyperkalemia. 

In addition, cardiac toxicity is a well-recognized complication of the administration of bupivacaine (and structural analogs) in both animals and humans [42–49]. Electrop-hysiological and hemodynamic disturbances, including conduction blocks, ventricular arrhythmias, and fatal CV collapse, have been reported in patients and observed experimentally in animal models. However, it is unclear whether the mechanism of death from bupivacaine toxicity is primarily a consequence of cardiac arrhythmias or of myocardial contractile depression, or some combination of the two. Some groups suggest that cardiotoxic bupivacaine concentrations produce a direct myocardial depression that precedes the onset of lethal arrhythmias. Others proposed that death from bupivacaine toxicity results from ventricular tachyarrhythmias, or severe bradycardia, with or without electromechanical dissociation, ultimately leading to CV collapse.

Rabbits have been reported to be more sensitive to the cardiotoxicity of bupivacaine than other animals [23]. It seems possible that a more rapid heart rate and reduced cardiac output may predispose to tissue accumulation of bupivacaine in the myocardium. In addition, tissue binding affinity (myocardium) and differing rate of metabolism play an important role. 

### 4.1. Data Interpretation

#### 4.1.1. Lack of Dose Response

In our studies, dogs tolerated much larger doses of EXPAREL than rabbits. A no-observable-adverse-effect level (NOAEL) dose for EXPAREL or Bsol was not achieved in rabbits. The tonic and/or clonic seizure activity seen with EXPAREL at 9 and 18 mg/kg as well with Bsol, although at lower frequency, were associated with bupivacaine and not the liposomal formulation. Complete recovery was observed after each dose indicating that these effects were reversible. 

It is our opinion that the major factors involved in the dramatic results seen in the rabbit compared to the dog were its susceptibility to bupivacaine. Under these stringent conditions, the test system was overwhelmed, which presumably contributed to the adverse effects. The exaggerated response achieved in rabbits was somewhat expected based on literature review, and, in some respect, mimics adverse reactions that could occur as a result of intravascular infusion and/or acute overdosing of bupivacaine. 

It is unclear why no convulsions were seen at the higher dose level of EXPAREL 30 mg/kg. Apparently, there is a toxicity threshold for concentration and exposure time, such that when surpassed, irreparable damage to target organs is produced. The lack of a clear dose response may reflect a differential expression of potassium channels, and, perhaps, compensatory mechanism(s) involving differential blockade and/or stimulation of excitory/inhibitory pathways, which could lead to a disproportional and nonlinear response.

#### 4.1.2. Potential Cause of Death

The day after receiving EXPAREL at a dosage of 30 mg/kg/dose given sc at biweekly intervals for a total dose of 30 mg/kg/dose × 6 injections = 180 mg/kg, one female rabbit was found dead. All other animals survived the duration of the study even those receiving a total dose 30 mg/kg/dose × 8 injections = 240 mg/kg in which the dose exposure was larger higher on a cumulative basis, and plasma concentrations were present for a longer period. 

The experimental conditions did not allow determination of the cause of death. It is possible that if this animal had been monitored constantly, earlier detection of delayed toxicity may have been possible. Intravascular injection is unlikely to have been an etiology in our case since the animal appeared normal on the day of dose administration. 

Considering the susceptibility of rabbits to cardiotoxicity and the fact that, after several repeat injections of EXPAREL, the compartments, being nearly saturated, may reach potentially toxic concentrations, led us to speculate that the lethality may have been caused by hypotension, respiratory distress, CV collapse, and/or sudden fatal ventricular tachycardia and fibrillation with or without hypoxia or acidosis. 

#### 4.1.3. Local Reactions

In recovery rabbits, the local reactions resolved to some degree, although minimal to mild HEM (hemorrhage), VMs, NV, and inflammation were present in few animals. The HEM, NV, and inflammation (chronic-active or subacute) seen in the EXPAREL-treated animals were possibly adverse effects although there was no clear evidence of a chronic response to EXPAREL consistent with a harmful response to the immune system. Some of the local inflammatory reactions may be caused by overt irritation produced by prolonged bupivacaine exposure [50–53]. There were occasional foci of GCs that surrounded exogenous basophilic mineralized material presumed to be DepoFoam or its breakdown products associated with chronic inflammation and mineralization of the exposed tissues or muscles. GCs, common inflammatory cells, are fused Macs (macrophages) partially resulting from the inability of Macs to phagocytize large particulates. This is a classic response that walls off and surrounds foreign material. These inflammatory defense mechanisms protect the body against entry of nontoxic foreign body particles. The presence of VMs appeared to resolve in a dose-dependent manner. The histiocytic infiltrate composed primarily of Macs in the reactive tissues (walls) likely indicate cellular uptake and processing of EXPAREL by local Macs. 

In dogs, the NOAEL was >30 mg/kg/dose. The low-grade Gi (minimal to moderate) observed with EXPAREL-dosed groups only decreased in severity, yet persisted in the recovery animals, and therefore was not considered reversible. The mineral deposits, commonly surrounded by GCs, were considered evidence of small amounts of foreign matter, presumably DepoFoam particles in the loose connective tissues of the sc space. With the low incidence and severity, these soft tissues changes are compatible with a foreign body type reaction following exposure to the tissue of the test article. The character of the soft tissue reaction was nonspecific and did not indicate any special toxic effect per se. In each species, the highest dose was administered via application of a concentrated formulation of EXPAREL (25 mg/mL); the formulation of 25 mg/mL was intended to maximize the delivery of EXPAREL to the site of absorption and was used to increase exposure of local tissues to relatively higher concentrations of both vehicle and drug. Despite the documented actions of bupivacaine on the musculoskeletal system, normal function of this system was not affected—even at both lipid and bupivacaine concentration 1.7 times higher than the undiluted EXPAREL formulation.

Notably, EXPAREL revealed a predictable sustained release profile in both species even at high doses. Notably, species difference was observed with lower *C *_max_ (↓4 fold) and *AUC* (↓5 fold) for all dose levels for EXPAREL (rabbit versus dog). The same observation was made for Bsol with lower *C *_max_(↓4–9 fold) and *AUC* (↓4 fold), perhaps because differences in tissue binding, vascular uptake, and hepatic clearance affect drug distribution. After repeat exposure, the modest accumulation of bupivacaine in rabbit plasma suggested that the highly concentrated formulation of EXPAREL was not cleared completely before the next dose was administered, as would be expected from its prolonged absorption from the injection sites. 

In contrast, dogs appeared to process bupivacaine similarly after the first dose and after the last dose; this finding is consistent with a lack of toxicity reported in this experimental model. The gradual input afforded by EXPAREL allowed enough time for the body to absorb bupivacaine and processed it without overwhelming the system even when massive doses were administered. 

In summary, we have identified a species difference as reflected in the greater incidence of local and systemic reactions in rabbits compared to dogs. In both species, EXPAREL was irritating to extravascular soft tissue when given in large amounts in excess of the clinical dosage. All microscopic changes at the injection sites were minimal to mild/moderate. Similar microscopic findings were not observed in Bsol or saline control group. In rabbits, the systemic reactions (tremors/convulsions) were attributed to an exaggerated response to bupivacaine and were more frequently observed with Bsol. As a result, a NOAEL was not identified in rabbits. The local reactions were expected consequences of EXPAREL, due to the deposition of foreign matter in the loose connective tissues of the subcutaneous space. 

#### 4.1.4. Clinical Relevance

The most important consideration in the findings from these studies is the potential implications on human risk. While the mechanism(s) that contributed to convulsions in this study cannot be identified with certainty, the toxicological effects of EXPAREL in rabbits, presumably, are a reflection of a low rate, threshold-sensitive phenomenon that is not operative, and/or anticipated in humans under actual condition of exposure (i.e., single dose). 

In the repeat-dose 28-day studies, the dosing methodology was selected to maximize exposure conditions. Under these conditions, the total cumulative dose of bupivacaine was regarded as excessive relative to the intended single-dose administration in the clinic, that is, the dosing regimen far exceeded the number of doses humans will receive. 

In dogs, no effects were noted. In rabbits, convulsions and one death were noted. The death was recorded in a female rabbit one day after receiving six injections of EXPAREL 30 mg/kg subcutaneously at biweekly intervals, which correspond to a total cumulative dose of 30 mg/kg × 6 doses = 180 mg/kg). Given the fact there was no dose-related response, the death may have been either incidental and/or related to excess responsiveness to bupivacaine action, that is, the lethality may have been caused by sudden fatal ventricular tachycardia and fibrillation leading to cardiac arrest as discussed above. 

There is no compelling evidence for this being due to the cumulative EXPAREL material per se that was injected. There is no evidence that the negative outcome in this animal is related to the specific formulation of bupivacaine used (EXPAREL) and/or the vehicle itself, but rather this extreme finding was considered to be incidental and/or most likely attributed to the sensitivity of this particular animal to the toxic effects of bupivacaine. 

The dog findings appear to be clinically more relevant than the rabbit, since humans usually do not experience severe effects unless very high doses of bupivacaine are given. However, caution must be emphasized since this may not be always the case. For example, patients with underlying pathology (e.g., renal failure, acidosis, or cirrhosis) may have higher sensitivity to the toxic effects of bupivacaine and structural analogs [54].

It is our opinion that the major factors involved in the dramatic results seen in the rabbit were due to physiol-ogical variations and species susceptibility to bupivacaine. Alteration in regional blood flow, hemodynamic instability, and a more rapid drug uptake along with a slow egress in target tissue may render rabbits more susceptible to drug accumulation and increase the risk of overt toxicity with prolonged administration of repeated doses. 

In summary, the nature and level of the findings in rabbits did not present a clinically significant safety concern since EXPAREL will be administered as a single-dose by local infiltration in a clinical setting. The relevance of the rabbit observations in terms of correlating to humans is limited, since this model uses excessive dosage relative to the intended clinical dose. The lack of significant toxicology effects in a second model may provide a higher level of comfort that EXPAREL does not pose a significant health risk especially after single dose administration. These studies however draw attention to the potential complications which may occur whenever bupivacaine in any form is used.

## 5. Conclusion

Taken together, the data demonstrate that rabbits are more susceptible to bupivacaine toxicity than dogs. EXPAREL was well tolerated in dogs during twice weekly administration for a total of 8 doses over the course of the study (cumulative NOAEL dose = 240 mg/kg). In this species, there was no indication of local or systemic complications over the course of the study. In contrast, a NOAEL was not identified in rabbits.

## Figures and Tables

**Table 1:** Incidence of convulsions following twice weekly subcutaneous injection of saline, EXPAREL 9, 18, and 30 mg/kg, and bupivacaine HCl solution 9 mg/kg to rabbits (3/sex/group).

Treatment	Dose (mg/kg)	Incidence of convulsions (clonic, unless noted)^a^							
		Week 1		Week 2		Week 3		Week 4	
		Dose 1	Dose 2	Dose 3	Dose 4	Dose 5	Dose 6	Dose 7	Dose8
Bupivacaine HCl^b^	9	—	—	3(M,) 1(F)	—	1(M) (tremors)	—	—	—

EXPAREL	9	1(M) (tonic)	—	—	—	—	—	—	—
	18	—	—	—	1(F) (tremors)	1(M) (tremors)	—	—	—
	30	—	—	—	—	—	—	—	—
Saline	0	—	—	—	—	—	—	—	—

^a^Number of animal affected on each dosing day; ^b^Sensorcaine M: male F: female; “—” No seizures recorded; ^c^Dose 1, 2, 3, 4, 5, 6, 7, 8 administered on Day 1, 4, 8, 11, 15, 18, 22, respectively.

Notes: On Day 1, one male rabbit receiving EXPAREL 9 mg/kg had seizures intermittently lasting for about 10 minutes. Tonic movements were characterized by prolonged and sustained convulsions started around 15 minutes postdose. On day 8, four animals receiving bupivacaine HCl solution (9 mg/kg) (3(M) and 1(F)) had convulsions intermittently for 20 minutes. Clonic movements were characterized by rapid succession of spasms and relaxation. The convulsions started around 15 minutes postdose and were less frequent with shorter duration as time went on. These animals recovered by 1 hour postdose. On Day 11, 1(F) receiving bupivacaine HCl solution 18 mg/kg had clonic convulsions/tremors intermittently for 5 minutes. This animal recovered by 40 minutes postdose. On Day 15, 1(M) Bsol 9 mg/kg and 1(M) EXPAREL 18 mg/kg had clonic convulsions/tremors.

**Table:** (a) Rabbit

Treatment	Bupivacaine (mg/kg/dose)	Accumulation ratio (Day 25 versus Day 1)			
		*AU* *C*_0–72 hr_(ng·hr/mL)	*C *_ max_(ng/mL)	*t *_1/2_(hr)	*t *_ max_(hr)

Bupivacaine HCl	9	0.88	1.02	1.17	1.07

EXPAREL	9	0.94	2.30	0.35	0.41
	18	1.55	2.36	0.36	1.40
	30	2.14	3.09	1.42	0.29

**Table:** (b) Dog

Treatment	Bupivacaine (mg/kg/dose)	Accumulation ratio (Day 25 versus Day 1)			
		*AU* *C*_0–72 hr_(ng·hr/mL)	*C *_ max_(ng/mL)	*t *_1/2_(hr)	*t *_ max_(hr)

Bupivacaine HCl	9	0.94	1.40	0.60	1.0

EXPAREL	9	1.90	2.46	0.61	1.0
	18	1.90	2.34	0.25	1.5
	30	1.71	1.44	1.38	0.26

**Table 3:** Mean pharmacokinetic parameters for bupivacaine in rabbits receiving twice weekly subcutaneous bolus doses of DepoFoam bupivacaine (EXPAREL) or bupivacaine HCl solution (mean ±SD; *N* = 3/sex/group).

Dosing day	Treatment	Bupi (mg/kg)	*AU* *C*_0–72 hr_ (ng·hr/mL)	*AU* *C*_0–72 hr_/Dose (ng·hr/mL)	*C *_ max_ (ng/mL)	*C *_ max_ /Dose (ng/mL)	*t *_1/2_ (hr)	*t *_ max_(hr)
1	Bupivacaine HCl	9	2,350 ± 587	261 ± 65.2	396 ± 315	44.1 ± 35.0	9.57 ± 3.10	2.50 ± 4.66
	EXPAREL	**9**	**2,520 ± 429**	**280 ± 47.6**	**213 ± 145**	**23.7 ± 16.1**	**42.0 ± 26.4**	**1.42 ± 1.39**
		18	3,750 ±503	208 ± 27.9	147± 59.6	8.18 ± 3.29	48.2 ± 32.4	3.33 ± 3.61
		30	4,710 ± 1150	157 ± 38.5	94.4 ± 45.2	3.15 ± 1.51	150 ± 78.8	26.3 ± 24.2

25	Bupivacaine HCl	**9**	**2,060 ± 454**	**228 ± 50.5**	**218 ± 122**	**24.3 ± 13.6**	**11.2 ± 4.76**	**2.67 ± 4.58**
	EXPAREL	9	2,380 ± 1170	264 ± 130	338 ± 136	37.5 ± 15.2	14.5 ± 9.25	0.580 ± 0.200
		18	5,810 ± 426	323 ± 23.7	347 ± 210	19.3 ± 11.7	17.2 ± 7.06	4.67 ± 9.47
		30^a^	10,100 ± 4590	336 ± 153	292 ± 233	9.73 ± 7.76	62.9 ± 42.6	7.50 ± 6.16

^a^One female died prior to Day 25.

**Table 4:** Mean pharmacokinetic parameters for bupivacaine in dogs receiving twice-weekly subcutaneous bolus doses of DepoFoam bupivacaine (EXPAREL) or bupivacaine HCl solution (mean ±SD; *N* = 3/sex/group).

Dosing day	Treatment	Bupi (mg/kg)	*AU* *C*_0–72 hr_ (ng·hr/mL)	*AU* *C*_0–72 hr_/dose (ng·hr/mL)	*C *_ max_(ng/mL)	*C *_ max_/dose (ng/mL)	*t *_1/2_^ a^ (hr)	*t *_ max_ (hr)
1	Bupivacaine HCl	9	9,720 ± 1,860	1,080 ± 207	1,420 ± 355	158 ± 39.5	16.9 ± 6.05	0.500 ± 0
	EXPAREL	**9**	**9,100 ± 4,460**	**1,010 ± 495**	**488 ± 335**	**54.2 ± 37.2**	**59.5 ± 49.1 ** ^**a**^	**0.500 ± 0**
		18	12,800 ±2,020	711± 112	560 ± 299	31.1 ± 16.6	104 ± 105	0.500 ± 0
		30	25,600 ±8,160	853± 272	633 ± 280	21.1 ± 9.34	31.8^*a*^	48.1± 30.2
25	Bupivacaine HCl	9	9,120 ± 4,090	1,010 ± 455	1,990 ± 304	221 ± 33.8	10.1 ± 8.54	0.500 ± 0
	EXPAREL	**9**	**17,300 ± 8,710**	**1,920 ± 968**	**1,200 ± 301**	**133 ± 33.4**	**36.2 ± 12.4**	**0.500 ± 0**
		18	24,300 ± 8,960	1,350 ± 498	1,310 ± 521	72.6 ± 28.9	25.7 ± 8.15	0.750 ± 0.610
		30	43,800± 23,300	1,460 ± 777	910 ± 433	30.3 ± 14.4	43.9 ± 12.7^a^	12.8 ± 18.0

^a^Parameter not calculated in one animal (9 mg/kg, Day 1), four animals (30 mg/kg, Day 1), and one animal (30 mg/kg, Day 25) due to insufficient number of data points during the terminal phase to estimate a reliable plasma half-life.

## Abbreviations

Bsol: Bupivacaine HCl solution
CNS: Central nervous system
CV: Cardiovascular
DEPC: Dierucoylphosphatidylcholine
EXPAREL (DB): Bupivacaine extended-release liposome injection using multivesicular DepoFoam technology
GCs: Multinucleated giant cells
HEM: Hemorrhage
iv: Intravenous
Macs: Macrophages
MPF: Methyl paraben free
NOAEL: No-observable adverse effect level
NV: Neovascularisation
PK: Pharmacokinetics
sc: Subcutaneous(ly)
SD: Standard deviation
VMs: Vacuolated (foamy) macrophages.
